# Maximization of container slot booking profits for carriers in the liner shipping industry

**DOI:** 10.1186/s41072-021-00100-7

**Published:** 2021-10-29

**Authors:** Yu Guo, Ran Yan, Hans Wang

**Affiliations:** Department of Logistics and Maritime Studies, The Hong Kong Polytechnic University, Hung Hom, Kowloon, Hong Kong China

**Keywords:** Liner shipping, Container slots booking, Newsvendor model

## Abstract

In the liner shipping industry, if a shipper wants to transport its cargo by container ships, it first needs to contact a carrier to book container slots based on the estimated transportation demand. However, one problem in the booking process is that the actual demand is uncertain, resulting in mismatch between the required demand and the booked quantity. To address this issue, this study develops a Newsvendor model to find the optimal order quantity of container slots for the shipper. In addition, uncertainties in the quantity of container slots booking made by the shipper might cause revenue loss to the carrier and low utilization of ship capacity in the daily operations of liner shipping services. Therefore, this study suggests that the shipper should pay reservation fee when booking container slots. This study also aims to find the maximum profit for the carrier under the optimal order quantity of the shipper. In sensitive analysis, how different prices per container slot offered by the carrier would influence the reservation fee, the optimal order quantity of the shipper, and the expected profit of the carrier are explored and discussed. This study can help to manage and promote the online container booking systems in the liner shipping industry.

## Introduction

Maritime transportation, which plays an important role in international trade (Psaraftis 2021), has two main operating modes: liner and charter. Liner transportation is a service of transporting goods, mostly containerized goods (containers), by ships with regular routes and fixed schedules. In contrast, ships involved in charter transportation do not have pre-established sailing routes and schedules as well as ports of call. This study aims to analyze the optimal order quantity of container slots within the context of liner shipping.

According to the data published by Alphaliner in August 2021, 6,254 container ships are in operation globally, with a total capacity of 24,938,712 twenty-foot equivalent units (TEUs) (Alphaliner TOP 100, 2021), which shows that there is a huge demand of container liner shipping transportation. Before actual transportation of goods, container slots booking is a main step. Slots on a container ship can be booked through an e-commerce platform. Several weeks before a voyage of a liner ship, its company, which is also called a carrier, will open the available container slots on that liner ship for online booking. If a shipper wants to transport his/her cargo to the destination port using liner shipping services, it first needs to contact the liner shipping company to book container slots through the e-commerce platform based on the estimated demand. Next, the shipping company accepts the booking and asks customer (i.e., shipper) to pack the cargo into the containers and deliver them to the container yard. Finally, the containers are loaded onto the liner ship and transported to the destination port.

Nowadays, e-commerce has provided an alternative way to launch shipping services, even in the relatively traditional maritime industry. For example, China has successfully used e-commerce in shipping and trade services to reduce delivery time (Panova et al. 2019). A series of shipping e-commerce platforms have also emerged in the shipping market. For example, Maersk launched its online ordering channel of container slots called Maersk Spot. More than 3000 shippers ordered slots on Maersk Spot, and the ordered quantity increased from over 50,000 forty-foot-equivalent units (FEUs) in the second quarter of 2019 (Wagner 2019) to 300,000 FEU in the fourth quarter of 2019 (Johnson 2020). Moreover, the “Internet + shipping” provides a new way to book container slots online, where liner shipping companies are trying to explore online sales channels to sell container slots (Lam and Zhang 2019). The development of 5G networks is expected to further enhance the efficiency of e-commerce platforms. In the liner shipping industry, e-commerce enables the traditional paper-based booking system to evolve into a more advanced online booking system. Two advantages can be offered by e-commerce platforms. First, shippers can efficiently access the newest available container slot information via the internet and make container booking plans according to their needs. Second, liner companies are able to obtain the up-to-date container reservation information and make corresponding adjustments in time. Thus, booking container slots online is beneficial to both shippers and carriers.

One problem in the booking process is that the number of slots ordered by shippers is based on estimation in most cases. However, the actual demand is uncertain as many factors, e.g., market environment and natural conditions, can impact the demand. As a result, the actual demand and the ordered quantity of containers may not match. More specifically, there are three cases of the demand uncertainty faced by a shipper. In the first case, the number of booked slots exceeds the actual demand; as a result, the shipper will cancel some slots. In the second case, the number of booked slots equals the actual demand. In the third case, the booked slots are fewer than the actual demand.

Consequently, the uncertainty brings risks to the carrier because the un-utilized container slots fail to generate revenue and thus cause revenue loss. Therefore, carriers should adopt a strategy to guarantee their profits. In this study, we assume that a shipper is required to pay a reservation fee when booking container slots and the remaining container fee will be paid after the cargo has been unloaded at the departure port. In other words, the reservation fee will become part of the transportation fee if the shipper successfully transports the estimated amount of cargo to the destination port. Meanwhile, the reservation fee will be non-refundable if the shipper cancels some or all the booked container slots.

## Literature review

Research papers of maximizing the carrier’s profit from container slots booking can be divided into two streams. One stream is focused on slot allocation, which is related to inventory control in revenue management. For example, Pei et al. (2007) developed a slot allocation model and proposed a method to calculate the allocation of containers according to different characteristics of ports and ships. Mao and Shen (2016) proposed a probability scheme-based slot allocation model for vehicular networks. Feng and Xiao (2006) developed a comprehensive decision support model to integrate decisions on pricing and allocation of container slots’ capacity. They found that the optimal decision was influenced by price, and strength of demand. Peng et al. (2017) proposed hybrid scheduling mechanisms that integrated the advantages of both distributed and centralized scheduling mechanisms. Atmayudha et al. (2021) stated that optimization is to select the best option of the type of ships (e.g., diesel or LNG) that carry crude oil to the refinery unit in each scenario. Lee et al. (2007) claimed that selling slots to the right customer with the right price at the right time could generate the maximum benefit for a liner company. Feng and Chang (2010) formulated a mathematical programming model to maximize the operational profit of slot allocation for ocean carriers, subject to constraints of vessel capacity, container demand, and empty container supply. Guo et al. (2018) established a stochastic allocation model considering the multiple dimensions of container type, size, deadweight tonnage, and capacity. This model was then combined with long-term contractual customer booking requirements and the randomness of supply and demand.

In practice, many factors can influence the demand for container slots in the shipping industry. Nevertheless, shippers book container slots based on the estimated demand in most cases, causing a possible mismatch between the actual and estimated container demands. As a result, shippers must cancel some or book more slots from the shipping market at a higher price. Therefore, another stream of container slot research is focused on the overbooking of container slots and analyzes the problem of container slot cancellation. For example, Wang et al. (2007) calculated the maximum expected profit and focused on the problem of overbooking in liner shipping. Later, Wang and Meng (2019) proposed three different forecasting models, i.e., a piecewise linear regression model, an autoregressive model, and an artificial neural network model, to predict the canceled quantities of container slots by mining container slot booking patterns from historical booking data. Unfortunately, due to the lack of real-time data, the up-to-date characteristics and patterns of container slot booking cancellation given the current shipping market conditions remain unclear. Schinas and Bergmann (2021) proved that Market‐Based Measures could be supported in international shipping under their findings of taxonomy. Zhao et al. (2019) proposed a conceptual model to analyze container slot cancellation in intercontinental shipping services between Asia and the US West Coast. Zhao et. al. (2020) further considered the primary factors influencing container slot cancellation to estimate the cancellation probability in long-haul transport in liner shipping services.

There are few existing studies aiming to determine the optimal number of container slots that a shipper should book. In the shipping market, booked container slots can be canceled for free, causing revenue loss to the carrier. In contrast, in the airline market, a customer pays a high cancellation fee if it cancels a booked seat due to personal reasons. Potentially, carriers can use a similar strategy to decrease the cancellation rate. Therefore, this study aims to determine the optimal order quantity of container slots for the shipper and the maximum profit for the carrier.

## Model

We use $q$ to denote a shipper’s real demand where $q$ is a random variable and has a uniform distribution in (0, 1). We use $x$ to denote the booked slots, and $x$ is a decision variable for the shipper and has a uniform distribution in (0, 1). The shipper’s demand is unknown; therefore, there are three cases of the actual and the booked quantity of container slots presented as follows.

(a) If $q<x$, the shipper books more container slots from the carrier than it really needs. Thus, the shipper needs to cancel $q-x$ slots. Note that the reservation fee is non-refundable in this case.

(b) If $q=x$, the number of booked slots matches the actual demand. As this case is rare in practice because the numbers of $q$ and $x$ are several tens or even several hundreds, we do not consider it in this study.

(c) If $q>x$, the shipper books fewer container slots from the carrier than it really needs. Therefore, the shipper needs to obtain $x-q$ more slots from the shipping market. In order to transport the cargo to the destination port on time, the shipper needs to find container slots in a limited time, and such urgent demand leads to the growth of the container slot booking fee as a consequence. Thus, the market price for a slot is higher than the price provided by the carrier (Sofreight 2020).

We further define some parameters to calculate the shipper’s cost. We use $\theta$ (USD) to represent the reservation fee of each slot booked. We assume that the transportation fee of each container slot is $\alpha$ (USD) offered by the carrier and $\alpha$ > $\theta$, and the market price of each slot is $\beta$ (USD). As the price for the $x$ container slots are decided by both the carrier and the shipper and is lower than the market price, we have $\beta$ > $\alpha$. Therefore, a shipper’s cost includes two parts, the reservation fee and transportation fee.

We start by calculating the cost of the shipper in the first case where $x > q$. In this case, the shipper books more container slots than the actual demand and the carrier only transports $q$ containers. Therefore, the total cost of the shipper includes the transportation fee for $q$ container slots and the reservation fee for $x-q$ container slots.

We denote by $C_{1}$ the total cost in the first case. The objective function can be formulated as follows:

1 $$C_{1} = \alpha q + \theta \left( {x - q} \right).$$

We then calculate the shipper’s cost in the case where $x < q$, which means that the shipper needs to book $q-x$ more container slots from the shipping market with price $\beta$. We denote by ${C}_{3}$ the total cost of the shipper in this case, and the total cost can be formulated as follows:

2 $$C_{3} = \alpha x + \beta \left( {q - x} \right).$$

### The shipper’s optimal order quantity

The problem of finding a shipper’s optimal order quantity is an instance of the newsvendor problem. The newsvendor or newsboy problem, also called a single-period inventory management problem, is an inventory management model that seeks to identify an optimal order quantity to maximize the expected profit in a period (Khouja 1999; Qin et al. 2011). The key insights stemming from the analysis of this newsvendor problem have a broad range of application in managing inventory decisions in many industries, such as hospitality, airline, and fashion goods. Therefore, this study applies the newsvendor model to determine the optimal order quantity of container slots for a shipper.

At the beginning of a single period, the shipper is interested in determining the optimal order quantity of container slots, denoted by ${x}^{*}$. The shipper’s booking demand is assumed to be stochastic and characterized by a random variable $x$ with the probability density function as $f(x)$ and the cumulative distribution function as $F(x)$. The carrier is assumed to operate with sufficient capacity and no capacity restrictions and zero lead time of supply. Therefore, the order placed by the shipper from the carrier at the beginning of a period is immediately fulfilled. The sales of the container slots occur during or at the end of a period. Thus, the actual cost at the end of the period for the shipper is

3 $$C=\left\{\begin{array}{c}\alpha q+\theta \left(x-q\right),q<x;\\ \alpha x+\beta \left(q-x\right), q>x.\end{array}\right.$$

As the demand is not realized at the beginning of the period, the shipper cannot observe the actual cost. Hence, a normal approach to analyzing the problem is to allow the shipper to make the optimal decision on ordering quantity at the beginning of the period to maximize the carrier’s expected total profit. Thus, the total expected profit for the carrier can be formulated as follows:

4 $$\begin{aligned} E\left( C \right) & = \mathop \smallint \limits_{0}^{x} [\alpha q + \theta \left( {x - q} \right)]f\left( q \right)dq + \mathop \smallint \limits_{x}^{1} \left[ {\alpha x + \beta \left( {q - x} \right)} \right]f\left( q \right)dq \\ & = \theta x + \beta + \left( {\beta - \alpha + \theta } \right)\mathop \smallint \limits_{0}^{x} qf\left( q \right)dq + \left( {\beta - \alpha + \theta } \right)\mathop \smallint \limits_{x}^{1} xf\left( q \right)dq \\ \end{aligned}$$

Next, by calculating the partial derivative of the expd profit for $x$, we obtain

5 $$\begin{aligned} \frac{\partial E\left( C \right)}{{\partial \theta }} & = \theta + \left( {\beta - \alpha + \theta } \right)\left[ {xf\left( x \right) + \mathop \smallint \limits_{x}^{1} f\left( q \right)dq + xf\left( x \right)} \right] \\ & = \theta + \left( {\beta - \alpha + \theta } \right)\left( {1 - x} \right). \\ \end{aligned}$$

Therefore, by setting $\partial E\left(C\right)/\partial \theta =0$, we can obtain the optimal order quantity ${x}^{*}$ as follow:

6 $${x}^{*}=\frac{\beta -\alpha }{\beta -\alpha +\theta }.$$

### The carrier’s maximum profit

In this study, we assume that the carrier’s marginal cost is 0, and thus the carrier’s profit equals the shipper’s cost. We denote $P$ by the carrier’s profit. As we have found the optimal order quantity ${x}^{*}$, we can use ${x}^{*}$ to substitute the carrier's profit function. Accordingly, we have

7 $$P=\left\{\begin{array}{c}\alpha q+\theta \left({x}^{*}-q\right), q<{ x}^{*};\\ \alpha {x}^{*}, q>{ x}^{*}.\end{array}\right.$$

We need to find the optimal values of $\theta$ and $\alpha$ to maximize the carrier’s profit. In doing so, we first find the carrier’s expected profit denoted by $E(P)$. Next, we can obtain the objective function as follows

8 $$\begin{aligned} E\left( P \right) & = \mathop \smallint \limits_{0}^{{ x^{*} }} [\alpha q + \theta \left( {x^{*} - q} \right)]dq + \mathop \smallint \limits_{{ x^{*} }}^{1} \alpha x^{*} dq \\ & = \alpha x^{*} - \frac{1}{2}\left( {\alpha - \theta } \right)x^{*2} \\ & = \frac{\beta - \alpha }{{2\left( {\beta - \alpha + \theta } \right)^{2} }}\left[ {\alpha \left( {\beta - \alpha } \right) + \theta \left( {\alpha + \beta } \right)} \right]. \\ \end{aligned}$$

Next, we calculate the partial derivative of the expected profit for $\theta$ as follows

9 $$\frac{\partial E(P)}{\partial \theta }= \frac{\left({\beta }^{2}-{\alpha }^{2}\right){\left(\beta -\alpha +\theta \right)}^{2}-2(\beta -\alpha +\theta )(\beta -\alpha )[\alpha \left(\beta -\alpha \right)+\theta (\alpha +\beta )]}{{(\beta -\alpha +\theta )}^{2}}.$$

The optimal ${\theta }^{*}$ can be found when $\partial E(P)/\partial \theta$ = 0, and we have the following equation under this condition

10 $$\left({\beta }^{2}-{\alpha }^{2}\right){\left(\beta -\alpha +\theta \right)}^{2}=2\left(\beta -\alpha +\theta \right)\left(\beta -\alpha \right)\left[\alpha \left(\beta -\alpha \right)+\theta \left(\alpha +\beta \right)\right].$$

After solving Eq. (10), we have

11 $${\theta }^{*}=\beta -\alpha -\frac{2\alpha \left(\beta -\alpha \right)}{\alpha +\beta }.$$

## Sensitive analysis

In this study, parameters such as the reservation fee, the price of container slots offered by the carrier, and the market price of container slots fluctuate in practice. In this section, we discuss how the reservation fee decided by the carrier and the expected profit of the carrier can be influenced by changing these parameters though sensitivity analysis. Sensitivity analysis aims to find out sensitive factors (e.g., the price of container slots offered by the carrier, and the market price of container slots) that have an important impact on the indicators (e.g., the optimal booking quantity for the shipper and the expected profit for the carrier), and then analyze and calculate the degree of influence on the indicators in the problem setting by changing the value of sensitive factors. Therefore, people can judge the risk tolerance of the problem from the results of sensitive analysis. One main shortcoming of sensitive analysis is that it cannot automatically determine the proper range of an uncertainty factor so as to consider the full range of uncertain situations and the probability of change within this range. Consequently, certain risks might be caused to the shipper and the carrier in our problem.

**Fig. 1:**
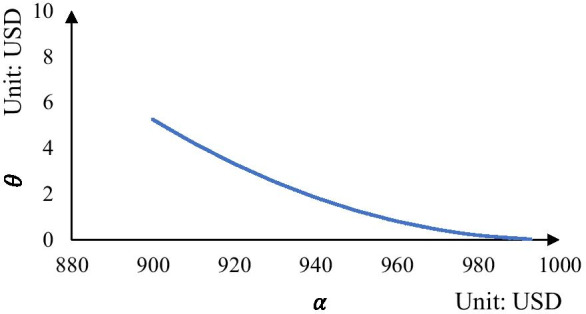
Optimal reservation fee under different values of $\alpha$

We first discuss how the reservation fee $(\theta )$ can be influenced by changing a container slot’s price offered by the carrier $(\alpha )$. We assume that the market price ($\beta )$ is equal to 1000 USD and set $\alpha$ in the range of $(0.9\beta ,\beta )$. The results are plotted in Fig. 1. It can be seen that the optimal reservation fee decreases and converges to 0 when the value of $\alpha$ increases and converges to $\beta$. The managerial insight generated for carriers is that a lower reservation fee should be set if the difference between $\alpha$ and $\beta$ is smaller.

**Fig. 2:**
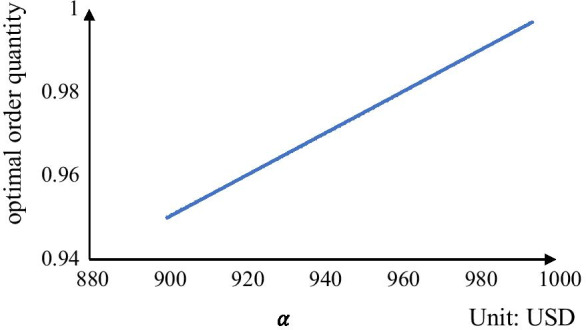
Optimal order quantity under different values of $\alpha$

Next, we analyze the impact of the price of one container slot offered by the carrier ($\alpha$) on the optimal order quantity ${x}^{*}$ for the shipper. We also set the market price ($\beta )$ at 1000 USD and change $\alpha$ from 900 to 1000 USD and the results are shown in Fig. 2. It indicates that the optimal order quantity of container slots increases when the price of one container slot offered by the carrier increases. This may be counterintuitive at first sight. However, a closer examination of Fig. 1 indicates that when the price of one container slot offered by the carrier increases, the reservation fee decreases, and their joint effect leads to the increase of the optimal order quantity.

**Fig. 3:**
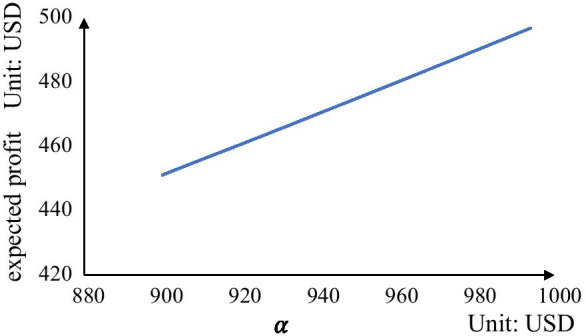
Expected profit under different values of $\alpha$

Finally, we consider how the expected profit of the carrier would be influenced by increasing the price of a slot offered by the carrier and the results are shown in Fig. 3. It can be seen that the carrier can obtain more profit when the value of $\alpha$ increases.

## Conclusions and future research directions

This study makes the first attempt to solve the uncertainty problem in container slots booking that causes risk to the shipper and the revenue loss to the carrier. We first propose a newsvendor problem to find the optimal order quantity of container slots for the shipper. We then calculate the carrier’s maximum profit under the shipper’s optimal order quantity of container slots. However, most parameters we consider fluctuate in practice. Hence, we assume that the market price is fixed and then discuss its impact on the reservation fee, the optimal order quantity, and the expected profit by increasing the price of one container slot offered by the carrier. This study can help to manage and promote the online container booking systems in the liner shipping industry.

Future studies will focus on the following aspects. First, this paper assumes that the carrier has sufficient capacity. However, there are many factors, e.g., the COVID-19, affecting the capacity of the carrier in actual liner shipping services. As a result, the capacity of container slots might be insufficient, which yields two cases of container slot booking for the shipper, i.e., the booked container slots of the shipper are either equal to or less than the carrier’s capacity. Furthermore, as affected by market factors, the actual demand is uncertain. Under this situation, finding the optimal order quantity of container slots while not exceeding the carrier's capacity is of highly significance for the shipper. Hence, future research can consider the optimal order quantity of the shipper under insufficient capacity of container slots.

Second, only one shipper is considered in this paper. As many shippers demand container slots from the shipping market, future research can consider more complex situations with one carrier and more shippers to find the maximum expected profit for the carrier while considering the shippers’ decisions. In the situation with one carrier and more shippers, two cases can be considered to calculate the maximum expected profit for the carrier. In the first case, all container slots in the capacity have been booked by those shippers. In the second case, there is surplus of the carrier’s capacity. Then, the carrier's expected profit should be calculated under each of the two cases.

## Data Availability

Not applicable.
